# Transcatheter Superior SVASD With PAPVR Repair Using Custom 8-cm Covered Stent and Superior Locking Technique

**DOI:** 10.1016/j.jaccas.2026.110019

**Published:** 2026-08-27

**Authors:** Randy S. Mohtar, Niall Linnane, Jamil A. Aboulhosn

**Affiliations:** a Department of Ecology and Evolutionary Biology, University of California-Los Angeles, Los Angeles, California, USA; b Ahmanson/UCLA Adult Congenital Heart Center, Los Angeles, California, USA

**Keywords:** atrial septal defect, computed tomography angiography, echocardiography, hemodynamics, right-sided catheterization, stents

## Abstract

**Background:**

Superior sinus venosus atrial septal defects (SVASDs) with partial anomalous pulmonary venous return (PAPVR) are complex interatrial communications typically requiring surgical repair. Transcatheter-based interventions are limited because of the lack of commercially available devices.

**Case Summary:**

A 54-year-old woman presented with dyspnea and palpitations. Multimodal imaging revealed a superior SVASD with PAPVR from the right upper/middle lobes to the superior vena cava/right atrium junction, causing a significant left-to-right shunt (Q_p_/Q_s_ of 1.8:1). A transcatheter closure was performed using a custom-manufactured 10 Zig 8-cm Cheatham-Platinum covered stent on a 22 mm × 8-cm balloon-in-balloon. A superior bare-metal anchoring stent was deployed to prevent inferior stent migration during flaring. Postprocedural hemodynamics confirmed significant shunt reduction (Q_p_/Q_s_ of 1.05:1).

**Discussion:**

Transcatheter closure of a superior SVASD with PAPVR in adults is technically feasible and favorable using custom-manufactured long devices.

**Take-Home Message:**

Tailoring custom-manufactured devices to patient anatomy provides a novel approach to personalized medicine for percutaneous SVASD/PAPVR repair.

## Visual Summary

**Figure:**
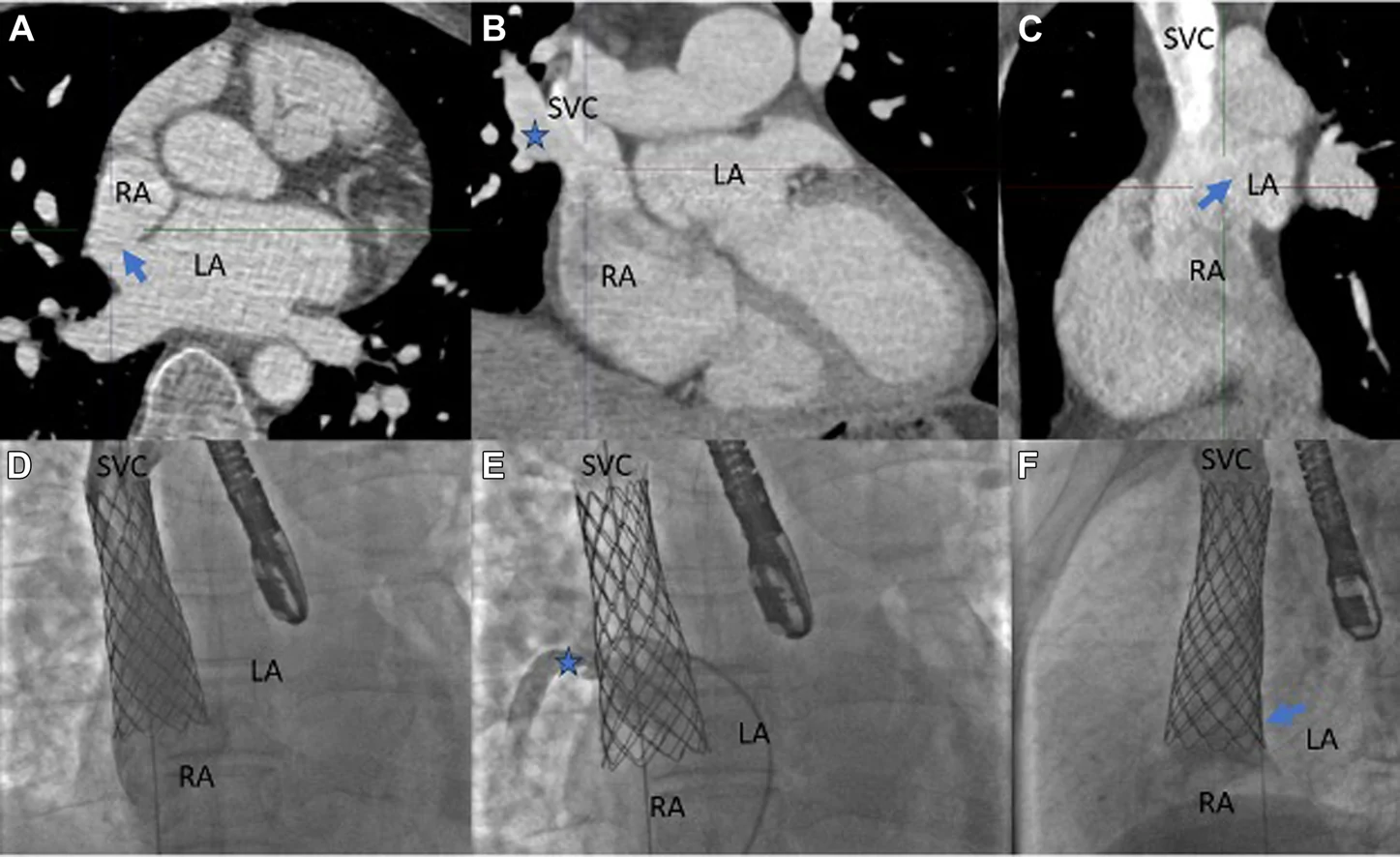

## History of Presentation

A 54-year-old woman presented with NYHA functional class III symptoms, specifically dyspnea on exertion and frequent palpitations. Initial electrocardiogram investigation at the emergency department suggested right ventricular hypertrophy with volume overload. The patient was referred to our adult congenital heart disease center for further hemodynamic and cardiac evaluation.

## Past Medical History

The patient's history is remarkable for an atrial septal defect diagnosis at 18 years of age following investigation of a heart murmur. At the time, no workup nor intervention was deemed necessary, given her asymptomatic status. She currently manages systemic hypertension with carvedilol and amlodipine. She has no history of arrhythmias or emboli.

## Differential Diagnosis

The patient's presentation was indicative of a substantial left-to-right shunt, often the result of a secundum atrial septal defect (ASD). Although secundum ASDs are the most common interatrial communication, imaging localized the defect superior to the fossa ovalis, effectively ruling out this possibility. The shunting may have been explained by independent partial anomalous pulmonary venous return (PAPVR); however, the defect visualized by computed tomography (CT) imaging made this unlikely (Figure 1A). Minor tricuspid valve regurgitation and pulmonary artery (PA) hypertension could explain the right ventricle (RV) dilation and diastolic septal flattening; however, cardiac catheterization revealed normal PA pressures. Given the superior location of the defect and associated PAPVR, a superior sinus venosus atrial septal defect (SVASD) diagnosis was likely.

**Figure 1:**
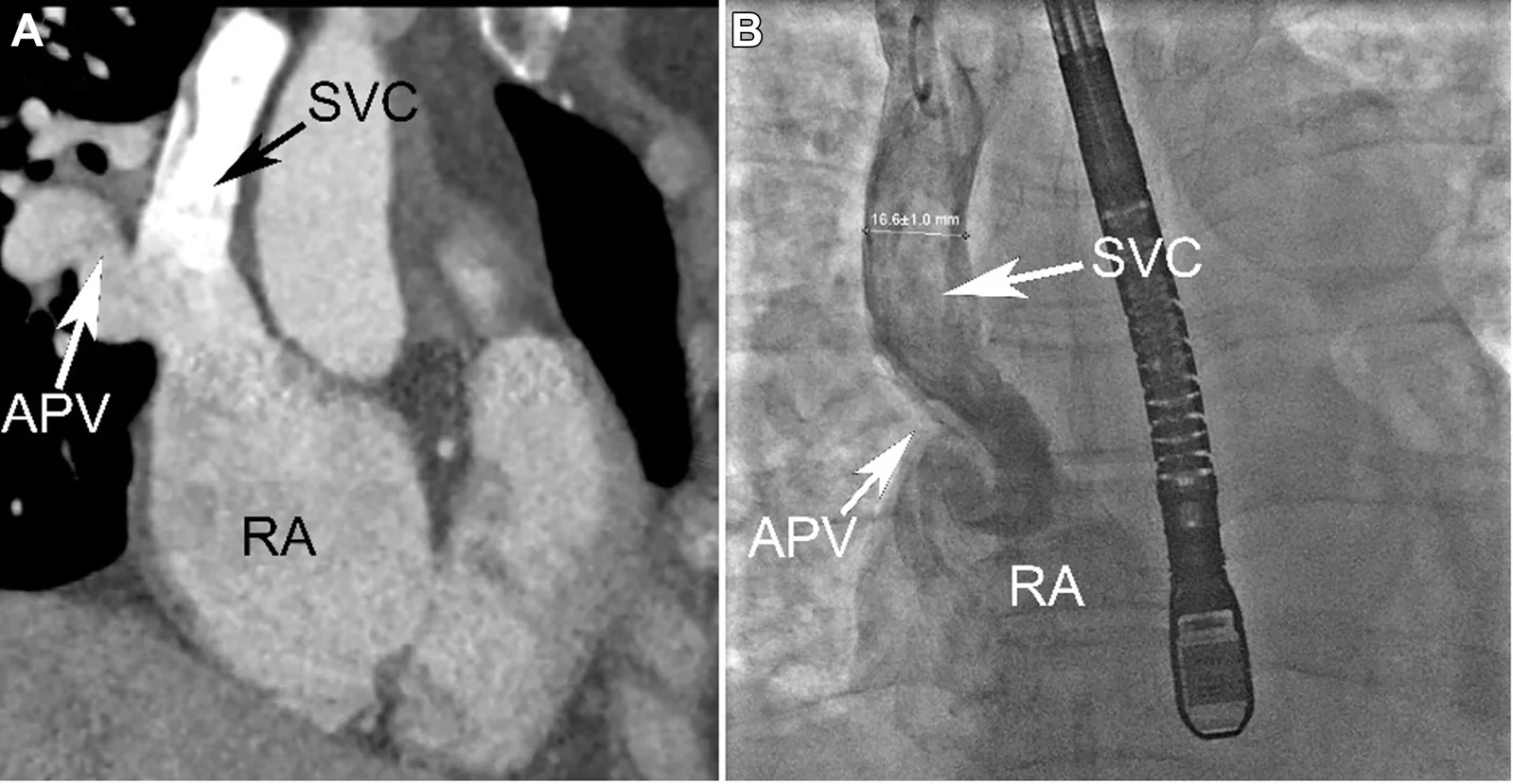
Preprocedural Heart Anatomy Detailing the Defect and Anomalous Flow (A) Computed tomography (CT) angiogram, coronal view, showing the superior vena cava (SVC). Note the anomalous pulmonary venous confluence (APV) entering at the SVC-to-right atrial (RA) junction. (B) Invasive cineangiography with contrast injection in the high SVC. Note the negative contrast effect of the flow from the APV into the SVC-to-RA junction.

## Investigations

Multimodal transesophageal echocardiogram (TEE) and transthoracic echocardiography confirmed a superior SVASD with associated PAPVR from the right upper and middle lobes to the superior vena cava (SVC)/right atrium (RA) junction (Video 1). CT angiography measured a 1.6-cm defect with main PA dilation (3.9 cm) (Figure 1B). Dilation of both the RV and RA was consistent with a large left-to-right shunt and volume overload. Diagnostic cardiac catheterization confirmed hemodynamically significant shunting with a Q_p_/Q_s_ of 1.8:1. Further cardiac CT imaging measured the high SVC at 18 × 19 mm and RA seal zone at 32 × 34 mm, estimating necessary stent coverage of 5.6 cm.

## Management

The patient met class I indications for closure of the defect by adult congenital heart disease guidelines.^1^ These include a Q_p_/Q_s_ ≥1.5, significant RV and RA dilation (volume overload), and pulmonary vascular resistance ≤2 WU. The complex anatomy of the superior SVASD led the outside institution to recommend open-heart surgery via median sternotomy. However, given the favorable landing zones and relative distance of the anomalous veins from the SVC-RA junction, our team elected to pursue a transcatheter approach using custom covered stents.

Fluoroscopy and TEE were used throughout the procedure to confirm anatomy and guide stent placement. Multivessel access was obtained via sheaths placed in the right internal jugular vein (7-F), right femoral vein (16-F exchanged for a 22-F 65-cm Gore DrySeal Flex Introducer Sheath [W.L. Gore & Associates]), left femoral vein (7-F), and right femoral artery (5-F). A transseptal puncture was made from the RA to the left atrium, allowing placement of an end-hole catheter in the anomalous pulmonary vein to assess for compression or obstruction. Balloon angioplasty of the SVC was performed using a 22-mm × 8-cm balloon-in-balloon (BIB) catheter (NuMED Inc); simultaneously, pulmonary venous pressure was measured, and a pulmonary venous angiogram was performed (Figure 2A). After confirming a patent pulmonary venous pathway, the balloon was deflated and withdrawn from the patient. Next, a custom-made 10 Zig 8-cm covered Cheatham-Platinum (CP) stent (NuMED Inc) was then mounted on a 22-mm × 8-cm BIB and positioned from the high SVC below the innominate vein down to the RA, covering the defect while preserving pulmonary venous drainage (Figure 2B). The stent was advanced via the right femoral vein and deployed under angiographic guidance from the right internal jugular vein (Video 2).

**Figure 2:**
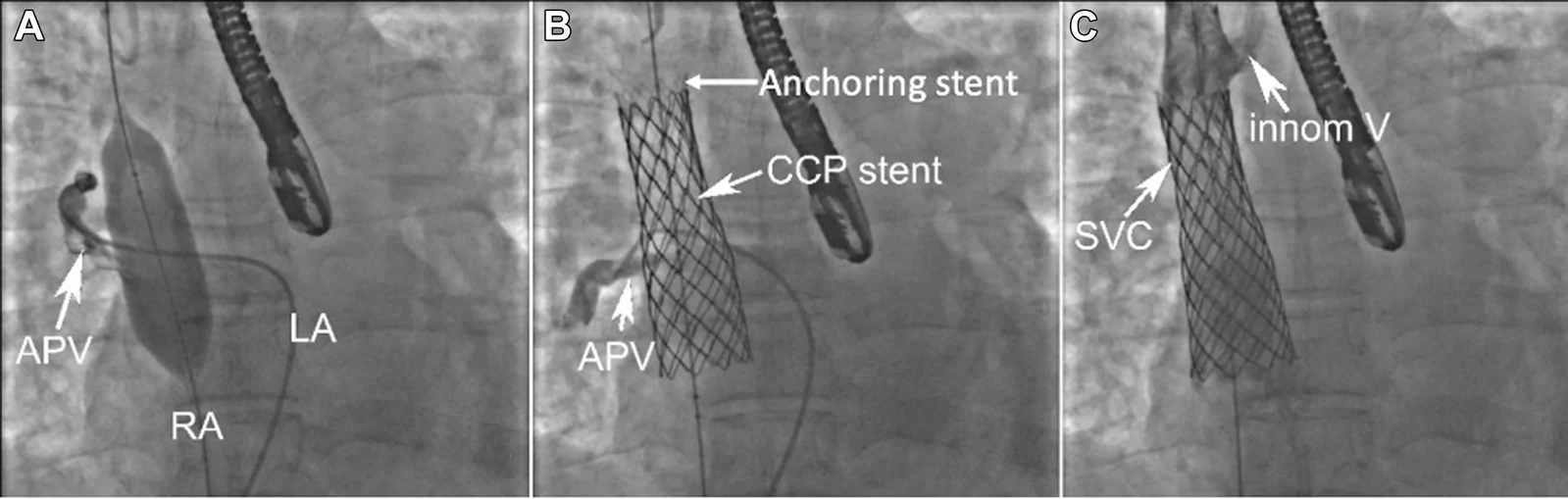
Balloon Sizing and Stent Placement in the Superior Vena Cava (SVC) to Right Atrial (RA) Junction (A) Balloon sizing of the SVC to RA junction with a 22-mm diameter balloon while simultaneously injecting contrast into the anomalous pulmonary venous (APV) to ensure patency of the channel from the APV to the left atrium (LA). (B) Contrast injection in the APV following placement of the covered Cheatham-Platinum (CCP) stent, demonstrating patent flow to the LA. Note the presence of the superior anchoring stent. (C) High SVC contrast injection demonstrating patency of the innominate vein and unobstructed flow through the CCP stent.

TEE demonstrated a small residual shunt through the SVASD; therefore, the decision was made to place a superior locking stent to stabilize the custom 8-cm covered CP stent. A 3110 Palmaz Genesis uncovered stent (Cordis) was mounted on a 24-mm × 4.5-cm BIB and deployed distally in the high SVC. The placement overlapped the CP stent superiorly while ensuring patency of the innominate vein. The proximal RA end of the stent was sequentially dilated with 24- and 30-mm diameter BIBs to flare the bottom and reduce residual shunting (Video 3). Intermittent sinus bradycardia occurred during balloon inflation and resolved with deflation, suggesting compression of the sinus node. TEE confirmed excellent stent positioning with minimal residual shunting and no evidence of pulmonary venous compression (Figure 2C, Video 4).

Repeat diagnostic catheterization at the end of the procedure confirmed reduction in the left-to-right shunting (Q_p_/Q_s_ reduced to 1.05:1) and expected mild left atrium pressure elevation (14 mm Hg).

## Outcome and Follow-Up

Short-term follow-ups (1-day to 2-week) confirmed stable stent positioning in the SVC with patent flow to the RA. TEE revealed significant reverse remodeling of the RV, returning to normal size and function (tricuspid annular plane systolic excursion 26 mm) with a normal systolic pressure of 30 mm Hg. No residual interatrial shunting was visualized by color Doppler, and a bubble study showed only faint, delayed left-to-right shunting, markedly improved compared to preprocedural measurements.

Six-day ambulatory rhythm monitoring revealed predominantly normal sinus rhythm with one brief, limited atrial tachycardic episode. By 4.5 months, the patient reported dramatic symptomatic improvement. Besides only occasional nighttime palpitations, she reported no syncope or volume overload, and increased exercise tolerance with comfortable ambulation. She remains clinically stable with planned periodic echocardiographic monitoring. She was placed on aspirin 81 mg daily to decrease the risk of stent thrombosis.

## Discussion

The current standard of care involves surgical closure of the superior sinus venosus defect coupled with anomalous pulmonary venous redirection via median sternotomy and cardiopulmonary bypass.^2^ Though transcatheter closure is an emerging alternative to surgical repair, the complex cardiac anatomy of a superior defect requires extended SVC-RA coverage that often surpasses the capabilities of commercially available CP covered stents, in which the longest foreshortens to approximately 4 cm.

In our patient, CT imaging necessitated approximately 5.5 cm of stent coverage. We partnered with NuMed Inc. to custom manufacture an 8-cm covered CP stent, accounting for approximately 2.5 cm (30%+) of stent foreshortening on distal flaring in the RA, allowing full coverage while avoiding potential risks associated with “telescoping” multiple stents.^2^^,^^3^ This approach represents the ultimate form of personalized medicine, requiring institutional review board and Food and Drug Administration compassionate-use authorization to create devices specific to the individual patient's anatomical needs.

Custom long covered stents are susceptible to inferior migration during dilation, prompting us to integrate a “superior locking” technique.^2^^,^^4^ A 3110 Palmaz Genesis uncovered stent was deployed distally into the high SVC, overlapping and anchoring the covered stent. This allowed for safe, sequential dilation of the RA end to reduce residual shunting, with a much lower risk of migration. Hemodynamically, our patient received significant shunt reduction, with a Q_p_/Q_s_ reduced from 1.8:1-1.05:1 and has shown favorable remodeling of the right heart. The 2.6 cm tricuspid annular plane systolic excursion and resolution of NYHA functional class III heart failure suggest that even in a 54-year-old, the right heart retains significant plastic potential for recovery after long-term volume overload.

The outcomes of transcatheter repair of a superior SVASD with PAPVR using long-covered stents are reassuring, with a low risk of serious complications. However, there is a learning curve to the procedure, and considerations must be made depending on the specific anatomy of the patient.^4^^,^^5^ Cross-sectional imaging with CT angiography or magnetic resonance imaging is needed for assessment of anatomy and estimation of needed stent length, as well as superior and inferior landing zone diameters. Delivering the large-diameter long stents into the SVC requires large-bore venous access; hence, techniques to minimize bleeding are often used (visual summary).^6^ Some operators have used self-expanding stent platforms as opposed to the balloon-expandable stent platform described in this report;^2^ regardless of stent type, appropriate preprocedural planning is indispensable, as is the operator understanding of stable stent placement without pulmonary vein compression.

In addition, balloon expansion and flaring the RA end of the stent can lead to SVC/RA or sinus node injuries, and must be managed carefully during the intervention.^7^ In the case presented, the diameter of the RA end flaring was limited to 30 mm given the intermittent loss of sinus rhythm and concern for permanent sinus node trauma. Moreover, the superior locking uncovered stent is deemed an important safety measure to stabilize the covered stent before inferior flaring. Effective and safe covered stent correction of the defect requires constant procedural imaging—in this case with TEE—to ensure appropriate ASD coverage and help assess for pulmonary venous obstruction.^8^ Postprocedural imaging and timely patient follow-up are important for assessing short- and long-term outcomes.

## Conclusions

Transcatheter repair of a superior SVASD with PAPVR using a custom-made 8 cm covered stent and superior anchoring stent is a technically achievable and favorable procedure.

**Take-Home Messages**

• Custom long-covered stents are often necessary to perform a transcatheter closure of an adult sinus venosus atrial septal defect to compensate for significant stent foreshortening.
• A superior uncovered locking stent placed in the high superior vena cava is an important addition to the repair to mitigate inferior stent migration during flaring.

**Visual Summary:**
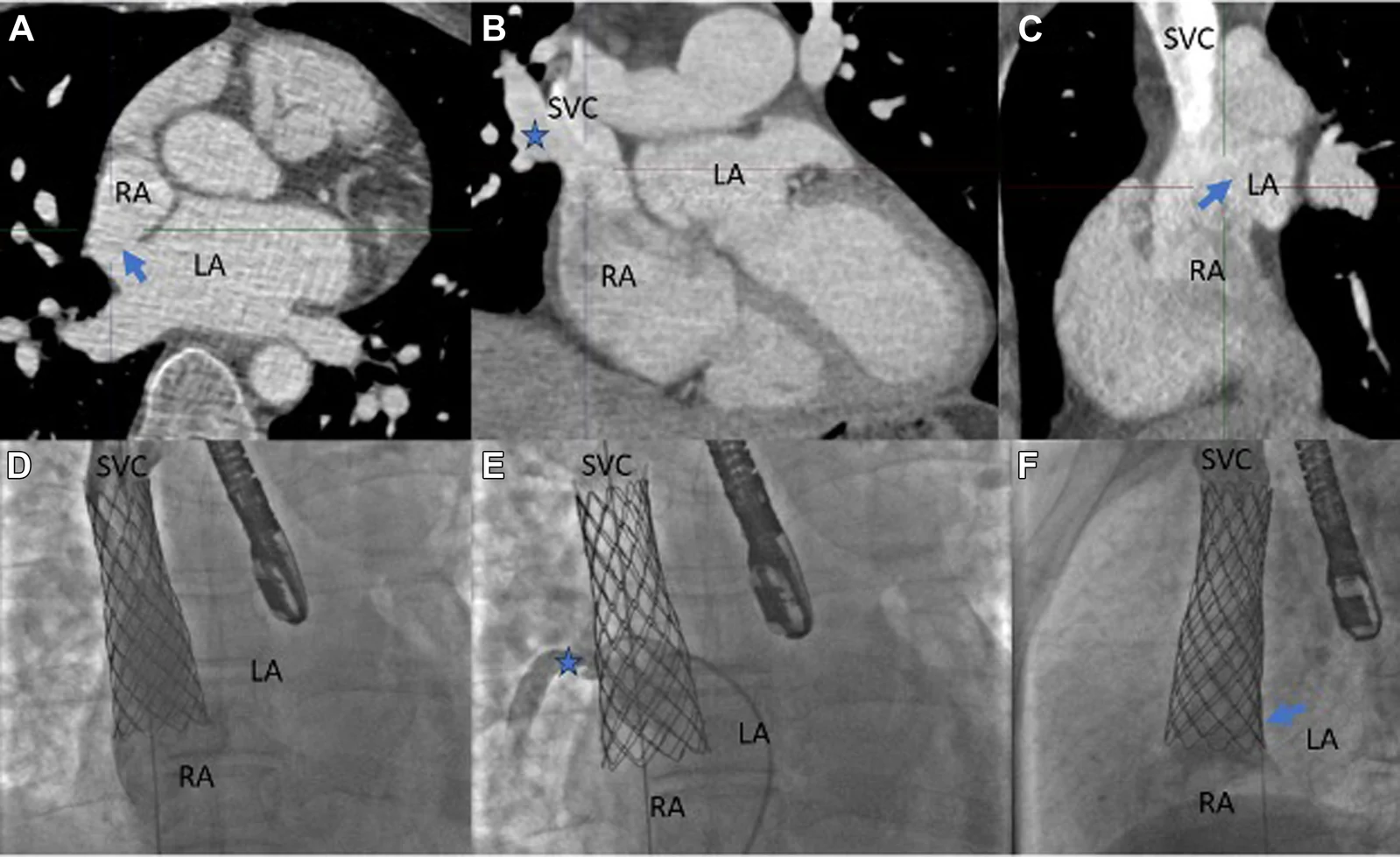
Collage of Images Highlighting the Preprocedural Anatomy by Computed Tomography Angiography (CTA) and the Intraprocedural Cineangiography After Covered Stent Placement (A) CTA axial view demonstrating the right atrium (RA), left atrium (LA), and the superior sinus venosus atrial septal defect (ASD) (blue arrow). (B) CTA coronal view demonstrating the anomalous right upper and right middle pulmonary venous connection (blue star) to the superior vena cava (SVC). (C) CTA sagittal view demonstrating the superior sinus venosus ASD (blue arrow). (D) Cineangiography, anteroposterior projection, with high SVC contrast injection demonstrating patency of the innominate vein and unobstructed flow through the covered stent. (E) Cineangiography, anteroposterior projection, with contrast injection in the right middle pulmonary vein (blue star). There is unobstructed flow to the LA and no contrast in the RA, consistent with occlusion of the superior sinus venosus ASD and unobstructed right-sided pulmonary venous return to the LA. (F) Cineangiography, lateral projection, with high SVC contrast injection demonstrating that the superior sinus venosus ASD (blue arrow) is covered by the stent, confirming patency and unobstructed flow.

## Funding Support and Author Disclosures

The authors have reported that they have no relationships relevant to the contents of this paper to disclose.

## Appendix

Video 1 Anomalous Right Middle Pulmonary Vein Angiogram Preintervention

Video 2 8-cm Long Covered Cheatham-Platinum Stent Deployment Right middle pulmonary vein angiogram reveals no compression.

Video 3 Inferior Flaring of the Covered Stent Following Superior Stabilization With the Anchoring Stent

Video 4 Right Middle Pulmonary Vein Angiogram Demonstrating Patent Drainage into the Left Atrium With No Residual Shunting.
